# Review of oncological emergencies in small animal patients

**DOI:** 10.1002/vms3.164

**Published:** 2019-03-21

**Authors:** Katrina L. Tumielewicz, Danielle Hudak, Jennifer Kim, David W. Hunley, Lisa A. Murphy

**Affiliations:** ^1^ Metropolitan Veterinary Associates Norristown Pennsylvania USA; ^2^ Cornell University College of Veterinary Medicine Ithaca New Jersey USA; ^3^ Northstar Vets Robbinsville New Jersey USA; ^4^ Gold Coast Center for Veterinary Care Huntington New York USA; ^5^ Veterinary Specialty Center of Delaware Newcastle Delaware USA

**Keywords:** oncology, hypercalcaemia, tumour lysis syndrome, chemotherapy, cancer, paraneoplastic, canine, feline

## Abstract

Oncological emergencies can occur at any time during the course of a malignancy and need to be recognized promptly to maximize successful outcomes. Emergencies are characterized as chemotherapy‐induced, paraneoplastic syndromes, or directly related to the neoplasm. Prompt identification with treatment of these emergencies can prolong survival and improve quality of life, even in the setting of terminal illness. This review aims to educate the reader on the pathophysiology, clinical presentation and treatment of some of these emergencies, and to review the current veterinary literature to help educate veterinarians in primary and tertiary facilities to know how to diagnose and treat these serious conditions.

## Introduction

The care of oncology patients, when they develop acute complications either from their underlying neoplasm or secondary to therapy, presents a challenge for veterinary oncologists, general practitioners and emergency clinicians. An oncological emergency is defined as an acute condition that is caused by cancer (paraneoplastic syndrome) or its treatment requiring rapid intervention to avoid death or severe permanent damage (Cervantes & Chirivella 2004). In this review, we will discuss the pathophysiology, presentation, diagnosis and treatment of commonly encountered emergencies in oncology with a focus on chemotherapy‐induced emergencies, gastrointestinal emergencies and acute paraneoplastic illness.

## Chemotherapy‐induced emergencies

Under the direction of a veterinary oncologist, chemotherapy administration is considered a relatively safe procedure with fewer than 25% of animals having adverse effects (AE) (Chun *et al*. 2001). A 2011 consensus document published by the Veterinary Cooperative Oncology Group (VCOG) provided practitioners with a grading scale for AE that can occur as well as the terminology to facilitate the accurate and consistent reporting of AE in patients receiving chemotherapy. Such standardization of terminology is essential for information transmission between clinicians and institutions (Yoon *et al*. 2007). These guidelines discuss a comprehensive range of AE and are divided into categories based on anatomy or pathophysiology with each given a grade of 1 through 5, from mild to death related AE. The reader is directed to the VCOG paper for further discussion of AE grading (VCOG 2011).

## Neutropenia

Neutrophils are the most abundant white blood cell in cats and dogs and are produced following release of granulocyte colony stimulating factor (G‐CSF) which stimulates neutrophil production. Neutrophil transit time through the bone marrow is 6 days and the circulating half‐life is 4 to 8 h (Lucroy 2005). In dogs, about half of the neutrophils in circulation are in the circulating pool and half in the marginated pool. In cats, only a quarter of neutrophils are in the circulating pool (Schultze 2010). Classification of neutropenia in dogs and cats differ with a value of less than 2500 cells *μ*L^−1^ in dogs and less than 2000 cells *μ*L^−1^ in cats considered neutropenic (Brown & Rogers 2001). However, in the authors’ experience, it is not common to see clinical signs related to neutropenia unless the neutrophil count is below 1000 cells *μ*L^−1^. Chemotherapy drugs are commonly associated with the development of neutropenia due to their myelosuppressive effects. In most instances of chemotherapy‐induced neutropenia, the patient is asymptomatic, so it may often go unnoticed or be found incidentally when a complete cell count (CBC) is rechecked prior to the next chemotherapy treatment. However, neutropenia can be accompanied by fever (temperature greater than 39.2°C), and clinical signs of infection may be present (Laforcade 2015). The true incidence of febrile neutropenia is not known but it is generally considered to be low. Risk factors for the development of neutropenia in dogs following chemotherapy administration include a lower body weight, dogs diagnosed with lymphosarcoma (LSA) and those who received doxorubicin (DOX) or vincristine (Brown & Rogers 2001; Sorenmo *et al*. 2010). With respect to cats developing neutropenia post chemotherapy administration, LSA was the most common diagnosis seen with both lomustine and vinca alkaloids associated with the development of neutropenia (Pierro *et al*. 2016).

**Figure 1:**
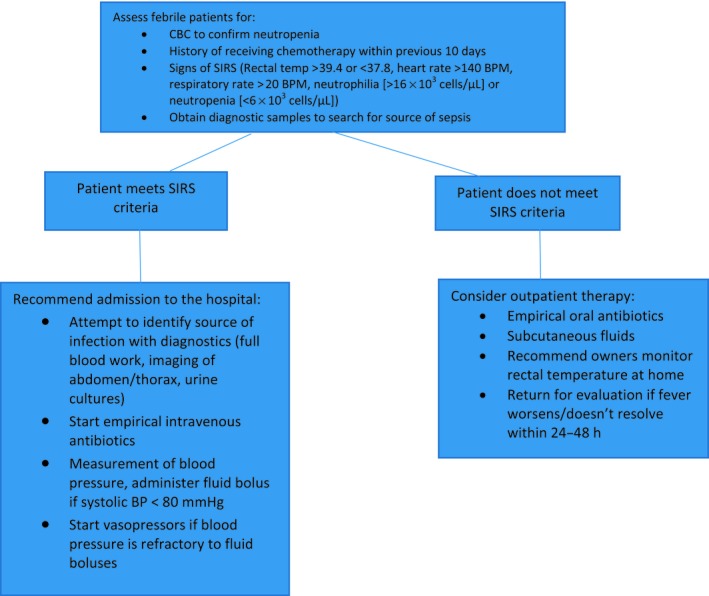
Management of febrile neutropenia.

Neutropenic patients may present to an emergency clinic with varying clinical signs, most commonly decreased appetite or lethargy after chemotherapy treatments. If these signs arise on or around the expected nadir of neutropenia of a recently‐administered chemotherapy drug (often 7–10 days after a chemotherapy treatment) then this is an indication that a bacterial infection may be present, especially if there is a concurrent fever (rectal temperature greater than 39.2°C). A CBC should be performed to confirm and determine the severity of the neutropenia. Patients with mild infections can often be treated on an outpatient basis, see Fig. 1.

Patients with more severe bacterial infections can present in systemic inflammatory response syndrome (SIRS) or in a state of sepsis. SIRS may resemble sepsis; however, it can occur in the absence of a documented infection (Brady & Otto 2001; Klainbart *et al*. 2017). For the definition of SIRS to be met veterinary patients must exhibit certain criteria, see Fig. 1, although such criteria are not considered 100% sensitive, however, the specificity can be quite high (Hauptman *et al*. 1997; Babyak & Sharp 2016). A recent study in a human emergency department also supported the use of SIRS criteria due to its high specificity and ease of use (Haydar *et al*. 2017). Sepsis is defined as a life‐threatening organ dysfunction caused by a dysregulated host response to infection (Singer *et al*. 2016). It has been noted in human literature that >50% of patients with febrile neutropenia or bacteraemia go on to develop sepsis (Penack *et al*. 2014). The incidence of sepsis from myelosuppressive chemotherapy in veterinary patients is relatively low and is likely multifactorial (Sorenmo *et al*. 2010). If left untreated, sepsis can be fatal.

The pathophysiology of sepsis remains incompletely understood but likely involves a combination of different cell types and a primary inflammatory stimulus under the influence of the innate immune system and T‐cells. The innate immune system responds to pathogens typically via receptors on the pathogen and a related ligand on an effector cell within the immune system. The major targets of these receptors are Pathogen‐associated molecular patterns (PAMPs) and Damage‐associated molecular patterns (DAMPs). The former responds to microbial pathogens while the latter respond to apoptotic cells. From a clinical standpoint, the end result of stimulation of either PAMPs or DAMPs is similar and once these receptors are stimulated, a cascade involving production of pro and anti‐inflammatory cytokines begins. Large production of proinflammatory cytokines is associated with the deleterious events observed in sepsis while an over‐production of anti‐inflammatory cytokines may lead to immunodeficiency and can also lead to a poor outcome (Pusterla *et al*. 2006; Briassoulis *et al*. 2010). Diagnostic measurement of cytokines may help physicians better characterize where in this pro or anti‐inflammatory cytokine spectrum individuals are falling however these are not widely available to most veterinarians at this time. Examples of such cytokines include TNF‐a which once released causes increased production of inducible nitric oxide synthase (iNOS) and cyclooxygenase‐2 (COX‐2), the former of which causes production of nitric oxide which can lead to hypotension. (Stylianou & Saklatvala 1998; Chaitanya *et al*. 2010). In contrast, COX‐2 will lead to significant inflammation. Other important cytokines produced include IL‐6 which triggers the acute phase response (APR) and is a potent pyrogen (Jawa *et al*. 2011). The APR is characterized by fever, activation of the coagulation and complement cascades and increased muscle and lipid metabolism (Cray *et al*. 2009). IL‐10 is the main anti‐inflammatory cytokine and acts to inhibit the release of other pro‐inflammatory cytokines and balance the pro and anti‐inflammatory processes occurring in sepsis. The end result of the release of such cytokines lead to multiple adverse effects across most of the body's organ systems and the clinical syndrome of sepsis.

For patients presenting septic, it is important to determine the source of infection where possible, in order to choose the most effective therapy. Diagnostic tests including imaging of the thorax and abdomen and/or blood and urine cultures can help identify the source of infection. Blood culture samples need to be obtained from different sites and should be obtained prior to starting antibiotics; however, this may not be feasible in all veterinary patients (Dellinger *et al*. 2012). Once necessary diagnostic samples have been taken, these patients should be started on broad spectrum antibiotics. Typically, empirical treatment using a beta lactam combined with a fluoroquinolone is pursued in small animal patients (Boothe 2015). Broad spectrum coverage to target both gram positive and negative microorganisms is recommended as gram negative organisms are the most commonly implicated infections in septic dogs and cats (Laforcade *et al*. 2003). Anaerobic coverage should also be considered for selected infections involving bone, deep or isolated areas and abscesses (Boothe 2015).

Sepsis remains a difficult syndrome to treat but improvements in the outcome of septic human patients have been accomplished with ‘bundle therapy,’ see Fig. 1. A bundle of therapy refers to therapies that, when instituted together, result in better outcomes than if each individual component were implemented alone (Cinel & Dellinger 2006). It is likely that a similar approach may improve outcomes in veterinary patients. One such bundle emphasizes the importance of measuring lactate within 6 hours of admission and reversing hyperlactataemia. Veterinary studies have found an improved prognosis with prompt lactate clearance (Bonczynski *et al*. 2003; Stevenson *et al*. 2007; Conti‐Patara *et al*. 2012). Other bundles recommend obtaining samples for culture or removal of the septic focus where possible and early administration of antibiotics, as previously discussed. The remaining bundles centre on the assessment and treatment of hypotension. Assessment of fluid responsiveness includes monitoring the animal's weight, vital parameters, blood pressure, mentation and urine output (UOP) via an indwelling urinary catheter. Finally, the guidelines also recommend the use of vasopressor agents in cases of refractory hypotension following fluid resuscitation. Noradrenaline is the first‐choice vasopressor in human medicine due to higher rates of adverse effects with adrenaline and dopamine (Puskarich 2012; De Backer *et al*. 2013, Vasu *et al*. 2012). Studies in veterinary medicine evaluating the superiority of various vasopressors are currently lacking. For clinicians managing septic patients, these can be quite labour‐intensive cases which typically require continuous monitoring and referral to speciality clinics is advisable to maximize success.

Myelosuppression and its secondary complications represent the major dose limiting toxicity of chemotherapy. Recombinant G‐CSF use in human oncology patients has been shown to redcue the severity and duration of neutropenia (Gabrilove *et al*. 1988; Morstyn *et al*. 1989; Trillet‐Lenoir *et al*. 1993). Clinical use of recombinant G‐CSF is limited in veterinary medicine due to its high cost in certain countries, the potential for auto‐antibody formation (Hammond *et al*. 1991), low incidence of febrile neutropenia, and lack of evidence that administration of G‐CSF will improve survival in cases of febrile neutropenia. Even severe neutropenia will generally resolve quickly secondary to production of endogenous G‐CSF (Britton *et al*. 2014), which is consistent with the authors’ experience. While there is currently not enough information to recommend the routine use of G‐CSF in veterinary patients, it should be noted that G‐CSF may be considered for patients in specialized circumstances where neutropenia may be severe and prolonged, such as those undergoing total body irradiation in combination with autologous peripheral blood progenitor cell transplants(, in cases of chemotherapy overdose and could be considered in difficult cases where the neutropenic chemotherapy patient is not responding to conventional therapy (Ogilvie *et al*. 1992; Escobar *et al*. 2012; Wells *et al*. 2014; Finlay *et al*. 2017).

## Anaphylactic reactions to Antineoplastic agents

Several medications commonly used by oncologists can induce anaphylaxis, including l‐asparaginase (L‐asp), DOX and drugs in the taxane family (Chun *et al*. 2001; Poirier *et al*. 2004; Blake *et al*. 2016, Axiak *et al*. 2011; Kim *et al*. 2015; Silva *et al*. 2015). L‐asp is an enzyme derived from Escherichia coli, all formulations are immunogenic since they are foreign proteins (Rogers 1989). DOX can induce hypersensitivity via stimulating histamine release (Susaneck 1983; Phillips *et al*. 1998). The exact mechanism for hypersensitivity from taxane chemotherapy drugs (paclitaxel, docetaxel) is unknown, but, there are several theories including complement system activation induced by Cremophor (which is compounded with paclitaxel) and polysorbate 80 (which is compounded with docetaxel). Stimulation of the complement system then activates mast cells. Recent use of a water‐soluble veterinary formulation of paclitaxel (Paccal^®^ Vet) without need for pre‐medication lends credence to this theory (Von Euler *et al*. 2013). Other theories include induced histamine release secondary to the drug's effects on basophils (Picard & Castells 2015).

Anaphylaxis can induce a range of acute clinical signs which can differ between species. In dogs dermal and gastrointestinal clinical signs predominate. The dermal signs are primarily secondary to the vasodilatory effects of histamine which causes erythema and pruritus. Many of the gastrointestinal signs occur secondary to hepatic congestion and portal hypertension which are also induced following histamine release leading to severe vomiting and diarrhoea (Shmuel & Cortes 2013). Gastrointestinal and respiratory tract signs are more typical in cats and they can present dyspneoic in severe cases (Shmuel & Cortes 2013; Kim *et al*. 2015; Murphy *et al*. 2016). Where anaphylaxis is suspected secondary to these drugs, administration should be stopped immediately. The animal should be treated with antihistamines, glucocorticoids and intravenous fluids to combat vasodilatory shock (Shmuel & Cortes 2013). However, the beneficial effects of glucocorticoids take several hours to occur and should never be considered the sole treatment for severe anaphylaxis (Shmuel & Cortes 2013). Furthermore, since histamine is just one of many vasodilatory substances released during anaphylaxis, anti‐histamines have limitations in animals with life‐threatening anaphylaxis. Intravenous fluids should be used judiciously in dyspnoeic animals; however, they should never be withheld as these patients are typically hypotensive either from hypovolaemic losses with vomiting and diarrhoea or have a relative hypovolaemia from vasodilatory shock. In severe cases where the blood pressure is refractory to the intravenous fluids and anti‐histamines, vasopressors are recommended. Adrenaline is considered the first line vasopressor for acute anaphylaxis by the World Allergy Association. There are no controlled trials in either veterinary or human medicine evaluating different vasopressors for anaphylaxis treatment. Such trials are considered potentially unethical because the preponderance of data in humans show that treatment with Adrenaline is critical for survival in many instances (Kemp *et al*. 2008; Shmuel & Cortes 2013). The beneficial effects of Adrenaline in cases of anaphylaxis stem from its effects on beta and alpha receptors. ß1 receptor agonism leads to positive inotropic effects increasing cardiac output. Stimulation of the ß1 receptor reduces release of inflammatory mediators by mast cells and has bronchodilatory effects (Shmuel & Cortes 2013). In addition, stimulation of alpha receptors by Adrenaline may help to alleviate laryngeal oedema and circumvent circulatory collapse via vasoconstriction (Estelle & Simons 2011). In dyspnoeic animals, supplemental oxygen should be provided. An inhaled bronchodilator can also be utilized to help relieve bronchospasm, but their use should not replace Adrenaline (Shmuel & Cortes 2013). After an anaphylactic event, the animal should not be administered the causative agent again. Although there is no way to completely prevent these reactions, some oncologists recommend the use of diphenhydramine and/or steroids as a premedication prior to the use of anti‐neoplastic agents that can induce anaphylaxis (Phillips *et al*. 1998; Silva *et al*. 2015).

## Extravasation

Many chemotherapeutic agents can induce significant tissue injury if extravasation (EV) of the drug occurs during administration. Examples of such vesicants include commonly used chemotherapy agents such as DOX and the vinca alkaloids (Villalobos 2006). The exact mechanism behind extravasation injury is not completely understood, but it is thought to be due, at least in part, to tissue damage secondary to free radical formation or damage to DNA. Progression of the tissue damage and subsequent necrosis occurs following diffusion of drug/DNA complexes. Non‐DNA binding agents such as the vinca alkaloids are metabolized quickly, allowing normal healing to occur after the initial injury, while DNA binding agents (such as DOX) will remain in the tissues and cause more prolonged injury (Dorr 1990).

**Figure 2:**
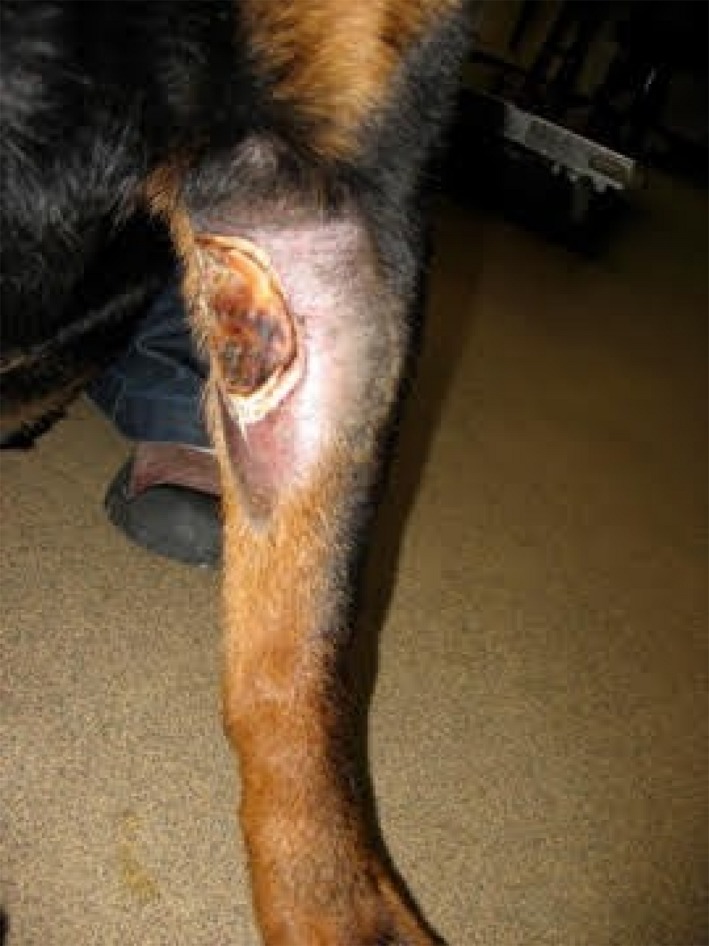
Extravasation secondary to doxorubicin in a dog.

Tissue necrosis typically is noticeable 1‐10 days after injection depending on the drug used. Severity ranges from erythema to more serious open draining wounds. Immediate treatment (Table 1) includes applying cold packs in the case of DOX and warm packs with the vinca alkaloids (Dorr 1990; Pattison 2002; Villalobos 2006). In cases of DOX EV (Fig. 2), treatments include dimethyl sulphoxide (DMSO) and hyaluronidase (HU) (Villalobos 2006; Mouridsen *et al*. 2007). More recently, dexrazoxane use has shown benefit in dogs and this is now the treatment of choice for treatment of DOX extravasation (Mouridsen *et al*. 2007; Venable *et al*. 2012). Dexrazoxane acts as a metal ion chelator protecting against free radical toxicity induced by DOX‐iron complexes (Langer *et al*. 2000). Ideally, dexrazoxane should be administered within 3–6 h of a known or suspected DOX EV and then again at 24 and 48 h. Use of dexrazoxane can be limited by its cost and availability. For EV associated with vinca alkaloids, treatments include infiltration of HU which promotes the diffusion of a chemotherapeutic by temporarily decreasing glycosaminoglycans which control diffusion of materials in and out of cells (Dorr 1990; Raszka *et al*. 1990; Pattison 2002; Spugnini 2002; Villalobos 2006). HU has been used successfully in cases of EV in several dogs given DOX and vinca alkaloids (Spugnini 2002). Steps to reduce the risk of EV occurring following chemotherapy administration include not performing blood draws on peripheral veins to preserve them for intravenous catheter placement (Villalobos 2006). Intravenous catheters being used for administration of these agents should have been placed recently (ideally within 4 h) and the catheter should be checked rigorously for patency with saline before and after the chemotherapeutic agent is given (Villalobos 2006).

**Table 1:** Treatment of extravasation

Drug	Intervention	Advice
DOX	DMSO	Apply locally immediately of a known/suspected DOX EV
	Dexrazoxane	Administer within 3–6 h of a known/suspected DOX EV
	Ice packs	Apply immediately of a known/suspected DOX EV
Vinca Alkaloids	Hyaluronidase	Instil 1 mL hyaluronidase (150 U mL^−1^) for every 1 mL of extravasated drug through the existing catheter line
	Hot packs	Apply immediately of a known/suspected vinca alkaloids EV

## Gastrointestinal toxicity

Although occurring less commonly in small animal patients compared to people, gastrointestinal toxicity (GIT) secondary to either the underlying cancer and/or chemotherapy can have a major impact on the patients’ quality of life. Risk factors for vomiting include administration of highly emetogenic chemotherapy, smaller patient size, repetitive administration of chemotherapy and a gastrointestinal comorbidity (Ogilvie *et al*. 1989; Moore *et al*. 1994; Vail 2009). There are two main causes of GIT in cancer patients: centrally mediated nausea and direct effects on the GI tract. The normal physiological pathway of vomiting is stimulated by several factors all of which converge on the vomiting centre within the medulla (Chapman 2015). This includes neural input via the vagal and sympathetic fibres secondary to gastrointestinal inflammation and/or over distension, anxiety/anticipation stimulating the cerebrum or excessive motion stimulating the vestibular centre. With respect to certain medications, including several chemotherapeutics, or toxins (e.g. uraemic, hepatic or endotoxins), these stimulate the chemoreceptor trigger zone (CTZ) which is a specialized region located within the fourth ventricle of the brain. This area is particularly sensitive to such substances because it lacks an intact blood brain barrier so emetic toxins and molecules can enter here relatively easily. Once stimulated, the receptors in the CTZ stimulate the vomiting centre in the brain. This area controls certain autonomic functions including swallowing, gastric sensation, baroreceptor function and pharyngeal sensation. Regardless of the exact stimulus, once the vomiting centre has been sufficiently stimulated, the process of vomiting begins (Chapman 2015). When caused by chemotherapy administration, centrally mediated nausea occurs acutely, either during the drug administration, or within the first few hours post‐treatment. This form of nausea is rarely an emergency and is generally controllable with injectable or oral antiemetics, which can be given prophylactically, prior to administration of a known emetogenic chemotherapeutic agent, or once signs of nausea are seen.

Decreased appetite, nausea, vomiting and diarrhoea commonly occur secondary to tumours that directly involve the GI tract or that cause external compression of the stomach or intestines. These signs occur secondary to GI obstruction, peritumoral inflammation, or affects GI motility and absorption. Ideally, treatment for this problem would be via effective treatment of the underlying cancer. This can be accomplished by surgical removal of the cancer or reducing tumour volume with other treatment modalities (e.g. chemotherapy, radiation therapy). Chemotherapy‐induced GIT can also occur secondary to the direct toxic effects of these drugs on the cells of the GI tract. Rapidly‐dividing multipotent stem cells in the intestinal crypts can be damaged by chemotherapy, resulting in depletion of enterocytes and villous blunting, which in turn leads to poor nutrient absorption (resulting in diarrhoea) and secretion of emetogenic factors (resulting in nausea and vomiting). These effects are often referred to as delayed GIT and generally occur 2–5 days after treatment, therefore if GI signs occur outside of this interval, it is important to consider other causes (unrelated to the chemotherapy) for these signs. The likelihood and severity of GIT post‐treatment varies with different chemotherapeutic agents.

Where vomiting is self‐limiting, animals can be fasted for several hours, although water should be provided. Once vomiting has ceased, a bland diet can be reintroduced (Vail 2009). Anti‐emetics can also be administered with the parenteral route preferred. Commonly used antiemetics include maropitant and ondansetron. For patients experiencing severe GI side effects, intravenous fluid therapy is recommended. A multimodal approach to antiemetic therapy may be required in these patients as their nausea can stem from both peripheral gastrointestinal tract dysfunction and stimulation at the central CTZ. For a full review of these anti‐emetics, the reader is directed elsewhere (Vail 2009; Whitehead *et al*. 2016).

## Tumour lysis syndrome

Tumour Lysis Syndrome (TLS) occurs when large numbers of tumour cells release their contents (nucleic acids, cations and anions including potassium and phosphorus) into the bloodstream simultaneously. TLS can occur spontaneously or more commonly in response to therapy as a result of rapid tumour cell death following effective chemotherapy treatment. Nucleic acids are initially broken down and release purines, adenosine and guanosine. Both are then further metabolized into inosine and guanine, respectively. The result of this pathway is the formation of hypoxanthine which itself is further broken down into xanthine and finally uric acid via the enzyme xanthine oxidase (Rigas *et al*. 1956; Hochberg & Cairo 2008; Howard *et al*. 2011). Uric acid, in particular, can have deleterious effects on numerous organ system, especially the renal system. It has several negative effects on the kidneys including renal vasoconstriction, impaired autoregulation and crystal‐induced tissue damage (Ejaz *et al*. 2007; Shimada *et al*. 2009). Based on the few case reports in the veterinary literature, the clinical ramifications of such changes usually result in acute kidney injury (AKI) in dogs and cats (Calia *et al*. 1996; Mylonakis *et al*. 2007; Vickery & Thamm 2007). Interestingly, most breeds of dogs, except English Bulldogs and Dalmatians, oxidise uric acid into allantoin in the liver via uricase (Page *et al*. 1986). Thus, hyperuricaemia may not be as important in renal injury in canine cases of TLS.

Two classifications of TLS have been developed in people (Cairo & Bishop 2004). Laboratory TLS is defined as two or more of the following metabolic abnormalities occurring up to seven days after initiation of chemotherapy: hyperuricaemia, hyperkalaemia, hyperphosphataemia and hypocalcaemia. However, clinical TLS is accompanied by an increased creatinine, seizures, cardiac arrhythmias and/or death. Specific classifications for dogs and cats with TLS are lacking in veterinary literature.

Literature evaluating the clinical signs of TLS in small animal patients is limited to several published case reports. One such report described the development of TLS in a dog several hours after radiation therapy (RT) for LSA (Vickery & Thamm 2007). Following RT, the dog developed vomiting and diarrhoea and on physical examination was noted to be tachycardic, tachypnoeic with weak femoral pulses on palpation. A similar report in a cat with TLS illustrated the development of shock several hours following RT for mediastinal LSA (Calia *et al*. 1996).

In human medicine the severity of TLS likely depends on the type of cancer, the cancer burden and patient characteristics (Howard *et al*. 2011). Known patient characteristics which confer a higher risk in humans include pre‐existing chronic renal insufficiency, oliguria, dehydration, hypotension and acidic urine (Howard *et al*. 2011). Such characteristics are not reported in veterinary medicine. The exact prevalence of TLS in cats and dogs is unknown. In the authors’ experience, however, TLS may also be inaccurately blamed for severe post‐chemotherapy illness that is unrelated to tumour cell lysis. If characteristic electrolyte derangements are not seen, or if there has been minimal reduction in tumour volume, it is important to look for other causes for the presenting clinical signs.

**Figure 3:**
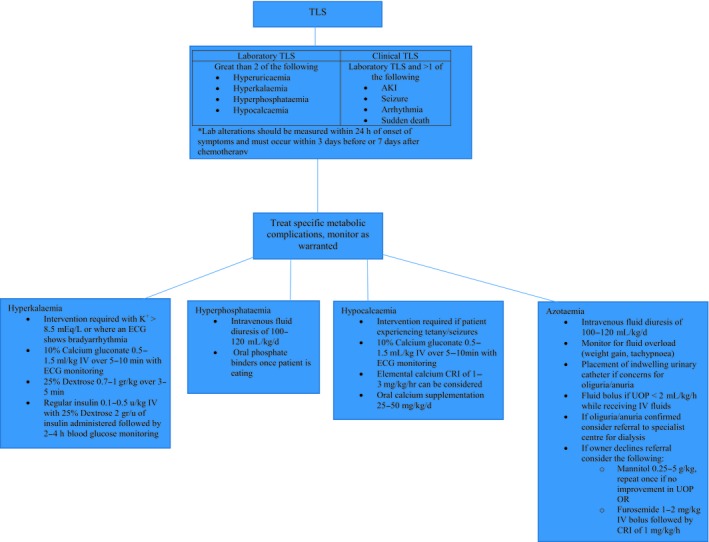
Management of tumor lysis syndrome.

Successful treatment of TLS depends on symptomatic management of the electrolyte disturbances and treatment of AKI, see Fig. 3. Current recommendations for management of AKI in humans include the discontinuation of nephrotoxic drugs, haemodynamic support with intravenous fluids, monitoring of UOP and, in cases of oliguria/anuria, vasoactive drugs (Keir & Kellum 2015). Once AKI is suspected, it is imperative that these patients are placed on a balanced intravenous fluid. An initial guide for prescribing intravenous fluids includes the following formula: percentage of dehydration x body weight in [kg] = fluid deficit in litres (Cavanagh *et al*. 2016). Even with cautious fluid administration, these patients are at risk of fluid overload which is typically defined as fluid accumulation more than 10% of baseline body weight (Cavanagh *et al*. 2016). Development of fluid overload and/or oliguria/anuria often indicates end‐stage AKI. Once this occurs, renal replacement therapy is considered gold standard for treatment; the prohibitive cost and availability affect its use in veterinary medicine. Vasodilatory medications have been studied for use in AKI as theoretically they can increase glomerular filtration rate (GFR) increasing UOP (Keir & Kellum 2015), see Table 2. When oliguria/anuria and/or fluid overload is suspected, the placement of an indwelling urinary catheter is recommended to measure UOP. Where the owner has declined referral for renal replacement therapy, it is recommended to trial the patient on a vasodilatory medication (see Table 2) in an attempt to increase UOP, although none of these medications have been proven superior. It is important to communicate to the owner that once oliguria/anuria occurs, without haemodialysis, there is a very low chance of conversion back to polyuria and the patient's prognosis at this point is grave.

**Table 2:** Vasoactive drug options for use in AKI

Vasoactive Drug	Drug Example	Dose	Comments	Adverse effects
Osmotic Diuretics	Mannitol	0.25–0.5 gr kg^−1^, do not exceed 2 gr kg^−1^	Dose can be repeated if no increased UOP noted within 30–60 min of administration	Volume overload, hyperosmolality
Thiazides	Hydrochlorothiazide	See comments	Generally these drugs are not potent enough to cause a significant increase in UOP	
Loop Diuretics	Furosemide	1–2 mg kg^−1^ loading dose followed by infusion of 1 mg kg^−1^ h^−1^ for up to 6 h	If UOP does not increase within 6 h discontinue, if UOP increases reduce to 0.1 mg kg^−1 ^h^−1^ to maintain urination	Dehydration, hypokalemia, metabolic alkalosis
Dopaminergic agonist	Dopamine	2–5 mcg kg^−1^min^−1^	The effectiveness of this drug has not been proven in human or veterinary medicine	Tachycardia, hypertension
DDopaminergic agonist	Fenoldopam	0.8 mcg kg^−1^min^−1^ (dogs) 0.5 mcg kg^−1^ min^−1^ (cats)	Studies in dogs/cats with acute kidney injury did not result in improved outcome	Hypotension

Prevention of TLS is considered imperative in human medicine but due to the multifactorial nature of TLS pathogenesis, it is difficult to make specific recommendations about preventative strategies in veterinary medicine. It is likely more important that veterinarians recognize the clinical signs of TLS as successful treatment depends on prompt diagnosis and appropriate treatment (Fig. 2). The prognosis for veterinary patients remains poor although more recent case reports show success in managing these cases (Calia *et al*. 1996; Vickery & Thamm 2007).

## Paraneoplastic syndromes

### Hypercalcaemia of malignancy

Hypercalcaemia (HC) is a common electrolyte derangement managed by small animal emergency clinicians. There are several possible causes of HC which can be categorized into non‐pathologic, transient and pathological groups; pathological causes are most common. Non‐pathological HC stems from normal physiological growth in young animals while hypoadrenocorticism or hyperproteinaemia can cause transient elevations. In contrast, pathological causes include neoplasia, primary hyperparathyroidism and acute or chronic renal failure. In canine patients, neoplasia is the most common cause of HC. In cats it is the third most common cause behind idiopathic causes and renal disease (Savary *et al*. 2000; Scheck *et al*. 2011). This review will focus on the paraneoplastic causes of HC of which several mechanisms are believed to be responsible for this phenomenon.

Paraneoplastic causes of HC include: ectopic production of parathyroid hormone (PTH) or PTH‐related peptide (PTH‐rP), extensive and usually multifocal lytic bone tumours, tumour associated prostaglandins (PGE1 and PGE2), interleukin 1‐ *β*, transforming growth factor –*β* (TGF‐ß) and receptor activator of nuclear factor kappa‐ *β* ligand (RANKL) (Bergman 2013).

Excessive production of parathyroid hormone related protein (PTH‐rP) is one of the most important causes of HC in the setting of neoplasia. Parathyroid hormone (PTH) is produced by the chief cells in the parathyroid gland and is involved in minute to minute control of calcium homeostasis in the body (Scheck *et al*. 2011). PTH‐rP is structurally and functionally related to PTH and is produced both in the normal physiological conditions and by certain types of cancerous cells (Scheck *et al*. 2011). Both PTH and PTH‐rP act on the same receptors and their effects consistently overlap. PTH‐rP stimulates osteoclastic bone resorption, increased renal tubular calcium reabsorption and decreased renal tubular phosphate reabsorption (Scheck *et al*. 2011). Other tumour‐produced factors which can also lead to HC include interleukin (IL)‐1a, IL‐6, transforming growth factor (TGF) and TNF alpha, these may act synergistically with PTH‐rP (Weir *et al*. 1988). However, measurement of these cytokines is not commonly performed and plasma PTH‐rP is the only assay that is readily available (Stern *et al*. 1985; Sabatini *et al*. 1988; Johnson *et al*. 1989). Common cancers associated with this phenomenon include T‐cell LSA and apocrine gland anal sac adenocarcinomas (AGASACA) (Williams *et al*. 2003; Bennett *et al*. 2002; Weir *et al*. 1988). Concurrent HC has been associated with a poorer prognosis in both LSA and AGASACA (Weir *et al*. 1988; Bennett *et al*. 2002; Williams *et al*. 2003).

Osteolytic metastasis and consequent excessive calcium release from bone also results in HC and is most often seen with carcinomas of the prostate, mammary gland, liver and lung (Rosol 2000; Meuten 1984). The pathogenesis of increased bone resorption is likely due to secretion of cytokines which stimulate local bone resorption and indirect stimulation of bone resorption from local immune or bone cells (Powell *et al*. 1991, Meuten 1984). Cytokines which may be involved include PTH‐rP, TGF alpha and beta and prostaglandins. Primary bone tumours are rarely associated with HC in dogs and cats (Quigley & Leedale 1983; Jongeward 1985; Mauldin *et al*. 1988).

Haematological malignancies in the bone marrow such as multiple myeloma and LSA also results in cytokine induced HC (Matus *et al*. 1986). Evaluation of animals with LSA show that HC in dogs is most commonly associated with the T‐cell phenotype (Weir *et al*. 1988; Rosol *et al*. 1992; Fournel‐Fleury *et al*. 2002). Studies in dogs and cats with multiple myeloma show approximately 20% of those with multiple myeloma develop HC. In dogs with multiple myeloma, concurrent HC is a poor prognostic indicator (Patel *et al*. 2005). It is believed that HC occurs with multiple myeloma due to increased osteoclastic induced bone resorption secondary to cytokines, tumour necrosis factor‐*β* and IL‐6, secreted locally by the myeloma cells (Oyajobi & Mundy 2004; Oyajobi 2007).

HC has wide‐reaching effects on many organ systems leading to a variety of clinical signs including polydipsia, polyuria, anorexia and constipation (Scheck *et al*. 2011). HC results in both functional and structural changes to the kidneys, the latter of which may not be reversible even with treatment. HC leads to renal arterial vasoconstriction reducing GFR. Subsequent azotaemia can be worsened by acute tubular necrosis from the toxic effects of calcium on renal epithelium, nephrocalcinosis and tubulointerstitial inflammation (Scheck *et al*. 2011). HC also reduces the excitability of GI smooth muscle, while parietal cells release hydrogen ions as a response to HC (Scheck *et al*. 2011). Clinically important cardiac effects of HC are uncommon in dogs and cats, but arrhythmias can occur from calcium's effects on myocardial cells or secondary to cardiac tissue mineralization (Scheck *et al*. 2011).

In animals symptomatic for HC, the calcium level should be normalized quickly to avoid organ damage although long‐term control of HC requires definitive treatment of the underlying cause. The best way to correct HC is to effectively treat the underlying cancer, either by surgical removal of the tumour, or by reducing the tumour volume with medical therapy (e.g. chemotherapy for LSA). If reduction in tumour volume is not possible, then the several treatment options can be considered to help control the animal's HC and secondary symptoms. Correction of dehydration with intravenous fluids is of paramount importance with 0.9% NaCl preferred as this promotes calciuresis via increased GFR (Scheck *et al*. 2011). Loop diuretics can be utilized following rehydration; thiazide diuretics are contraindicated as they can worsen HC (Duarte *et al*. 1971). Steroids can also be utilized to reduce calcium via decreased bone and intestinal calcium resorption and increased renal calcium excretion (Peterson & Feinman 1982). Steroid administration can prevent diagnosis of LSA and must be used judiciously and only after diagnostic samples have been obtained. Calcitonin can have rapid effects, often within hours of administration, on calcium by reducing the activity and formation of osteoclasts, making it an attractive choice for the emergent treatment of HC (Dougherty *et al*. 1990; Scheck *et al*. 2011). Following administration, animals must be monitored for development of hypocalcaemia as its effects on reducing calcium are profound. Bisphosphonates are commonly used in veterinary medicine for chronic management of hypercalcaemia associated with osteolysis or in patients with HC and a gross tumour that is not responding to treatment (or that the owner has decided not to treat). They act by decreasing osteoclast activity. Calcium levels typically take 2–5 days to decrease and effects last for 4–6 weeks (Scheck *et al*. 2011). Possible adverse effects include oesophageal and gastrointestinal toxicity with oral administration and electrolyte abnormalities (Morden *et al*. 2015). There are reports of nephrotoxic effects from bisphosphonates, therefore ideally, they would only be used in a well‐hydrated patient, although no significant adverse effects were noted with its use in one cohort of dogs (Machado & Flombaum 1996; Fan *et al*. 2005). Rapid administration of IV pamidronate can increase its nephrotoxic potential so it is recommended that it be given at a rate of less than 200 mg h^−1^ (Adami & Zamberlan 1996). Caution should be taken with intravenous bisphosphonate administration as it can be a tissue irritant if EV of the drug occurs. It is important to educate the owner that while there are several treatment options for HC, without controlling the underlying cause it is difficult to maintain normocalcaemia.

### Hypoglycaemia

Insulinoma is the most common malignancy associated with hypoglycaemia (HG); however, it has been reported with non‐islet cell tumours including hepatocellular carcinoma, hepatoma, LSA, leiomyosarcoma, adenocarcinoma and haemangiosarcoma among others (Patnaik *et al*. 1981; Cohen *et al*. 2003; Liptak *et al*. 2004; Battaglia *et al*. 2005). An insulinoma will produce excessive quantities of insulin leading to secondary HG as glucose is translocated into the cells. Theories postulated for how other malignancies can induce HG include secretion of insulin‐like substances, accelerated glucose utilization by the tumour and a failure of hepatic gluconeogenesis or glycogenolysis (Finora 2003). Animals experiencing HG often exhibit neurological clinical signs including weakness, disorientation, seizures or obtundation. In cases of insulinoma, a diagnosis of the HG can be ascertained via an insulin glucose ratio (Leifer *et al*. 1985). However, conditions other than insulinomas can yield increased ratios, and cases with confirmed insulinoma can still have normal insulin: glucose ratio making the diagnosis difficult (Thompson *et al*. 1995; Fernandez *et al*. 2009). In the case of extra‐pancreatic tumours, it can also be difficult to diagnose the exact cause of the HG. In the acute setting, HG patients are stabilized with a continuous infusion of dextrose. Medications, including glucocorticoids, glucagon and beta blockers, can also be used to promote normoglycaemia while more investigations and definitive treatments are pursued (Smith *et al*. 2000; Datte *et al*. 2016).

## Neoplasia‐related emergencies

### Haemorrhagic effusions

In some cases, animals may present acutely with clinical signs secondary to haemorrhagic effusion from the tumour. This review will focus on pericardial and peritoneal haemorrhagic effusions related to neoplasia. Less commonly, spontaneous haemorrhagic pleural effusion can also be associated with neoplasia; however, readers are directed elsewhere for information regarding this (Nakamura *et al*. 2008).

### Pericardial effusions

Neoplasia is the most common cause of pericardial effusion in dogs, with the right atrium and heart base being the most common sites (Ware & Hopper 1999; Vicari *et al*. 2001; Ehrhart *et al*. 2002; Johnson *et al*. 2004). On histology, the most commonly identified neoplasm is haemangiosarcoma (HSA), but others have been reported, including chemodectomas, other neuroendocrine tumours, ectopic thyroid carcinoma, mesothelioma and LSA (Ware & Hopper 1999; MacDonald *et al*. 2009). Many studies of neoplastic PE in dogs are based on cardiac HSA due to its predominance. The most common presenting signs noted in dogs include muffled heart sounds, tachypnoea, weakness and exercise intolerance (Johnson *et al*. 2004). Many of these patients may exhibit abnormalities on an electrocardiograph (ECG) including electrical alternans and ventricular arrhythmias (Johnson *et al*. 2004). PE can also lead to the development of pericardial tamponade (PT), which if untreated is fatal. PT occurs when the intra‐pericardial pressure increases from its normal sub‐atmospheric pressure and becomes closer to the right atrial pressure, (Reed & Thomas 1984). Following PT, venous return, ventricular filling, stroke volume and cardiac output decrease. Initially, these patients compensate with increased heart rate and peripheral vascular resistance, but as left ventricular filling becomes more compromised cardiogenic shock ensues (Reed & Thomas 1984; Bouvy & Bjorling 1991). Patients presenting with PT exhibit weak peripheral pulses, jugular venous distention or a positive hepatojugular reflux, laboured breathing, ascites and vomiting (Ware 2015; Fahey *et al*. 2017). Pulsus paradoxus (PP) may also be found upon physical examination of these patients. On palpation of femoral pulses in patients with PP, weak pulses are noted during inspiration followed by a stronger pulse during expiration. Normally during inspiration, the right side of the heart fills and the interventricular septum (IVS) is shifted leftward to allow for the increased volume. However, in cases of PE, left ventricular (LV) filling has already been limited by the pressure created by the effusion on the heart wall. The combination of the normal physiological IVS shift during inspiration and the pathological reduction in LV filling from the PE result in reduced stroke volume during inspiration and a weaker femoral pulse on palpation. During expiration the thoracic pressure changes from negative to positive allowing improved LV filling and a palpably stronger femoral pulse. PP will not be present in all cases of PE and it should be noted that in human medicine it can also occur in cases of constructive pericarditis, pulmonary embolism, right ventricular infarct, severe hypovolaemia and circulatory shock, bilateral pleural effusion, tension pneumothorax, chronic obstructive pulmonary disease, extraneous cardiac compression and tracheal compression (Swami & Spodick 2003; Argulian & Messerli 2013). PP is best appreciated in dogs which are in lateral recumbency (Shaw & Rush 2007).

Echocardiography is a commonly used test for diagnosis of cardiac masses in dogs with PE and for PT. When performed by a board‐certified cardiologist this diagnostic test boasts a sensitivity between 82–99% to identify cardiac masses (MacDonald *et al*. 2009). Cardiac HSA are most commonly located within the right atrium and/or right auricle. Uncommonly, they can extend through the atrioventricular junction, septum, the ventricular wall and rarely into the heart base (MacDonald *et al*. 2009; Yamamoto *et al*. 2013). Ultrasound guided aspirates of a cardiac mass may be performed to ascertain a cytological diagnosis although because many of these tumours do not readily exfoliate, these samples may often be non‐diagnostic. In one study in six dogs with different causes of PE the procedure did not result in major complications (Pedro *et al*. 2016). The use of fluid cytology to obtain a diagnosis can also have mixed results depending on the underlying aetiology and is generally recommended to only submit when the effusion has a low haematocrit (Cagle *et al*. 2014). In such cases, where the effusion has a low haematocrit, it may be more likely to be due to a non‐neoplastic cause and clinicians should consider inflammatory and infectious disease differentials. A study evaluating the utility of fluid cytology to identify the cause of pericardial effusion noted that only 6 of 47 dogs had a cause identified (MacDonald *et al*. 2009). Of those 5/6 were due to infective pericarditis and 1/6 dog had lymphoma.

Primary treatment involves removal of the fluid via pericardiocentesis. Arrhythmias, particularly ventricular arrhythmias, are the most common complication and occur in approximately 10% of dogs within one hour of the procedure and in 15% within forty‐eight hours (Humm *et al*. 2009). Humm found in dogs that develop such events, 41% were euthanized or died within forty‐eight hours of diagnosis with the majority being suspected to have cardiac HSA (Humm *et al*. 2009). Unfortunately, in cases of HSA, PE likely recurs, and average survival time is consistently less than 30 days (Dunning *et al*. 1998; Ware & Hopper 1999; Johnson *et al*. 2004; MacDonald *et al*. 2009). While many treatments for cardiac HSA have been evaluated, survival times remain poor (Kerstetter *et al*. 1997; Jackson *et al*. 1999; Weisse *et al*. 2005; Ghaffari *et al*. 2014; Mullin *et al*. 2016). Reported survival times for pericardectomy alone was 52 days while mass resection alone or with chemotherapy resulted in a mean survival time of 46 and 164 days respectively (Kerstetter *et al*. 1997; Weisse *et al*. 2005). Prognosis can be better with other types of neoplasia depending on the tumour type and treatment pursued. Of the heart base tumours, mean survival after diagnosis of chemodectoma in dogs was over 200 days although the use of pericardiectomy can improve mean survival time further (Vicari *et al*. 2001; Ehrhart *et al*. 2002). Cardiac LSA can also result in PE. Survival with adjuvant chemotherapy was over 150 days in one study (MacGregor *et al*. 2005). Without chemotherapy, survival is similar to that of cardiac HSA (MacGregor *et al*. 2005).

### Peritoneal effusions

Similar to PE, spontaneous haemorrhagic peritoneal effusion is a common syndrome noted in small animal practice. For dogs and cats presenting with haemorrhagic peritoneal effusion, malignancy is the most common cause, usually secondary to splenic ruptures (Mandell & Drobatz 1995; Pintar *et al*. 2003). HSA is the most common tumour associated with splenic neoplasia in dogs, accounting for 45–51% of splenic malignancies (Spangler & Culbertson 1992; Day *et al*. 1995; Spangler & Kass 1997; Hammond & Pesillo‐Crosby 2008). Dogs presenting with a non‐traumatic haemoperitoneum and a splenic mass have a 62‐70% chance of having HSA (Pintar *et al*. 2003; Hammond & Pesillo‐Crosby 2008; Aronsohn *et al*. 2009). In cats, causes of spontaneous haemoperitoneum are evenly distributed between neoplastic and non‐neoplastic causes with HSA being the most common neoplasm (Culp *et al*. 2010).

Presenting complaints of haemoperitoneum can be vague and include weakness, ataxia, abdominal distension, collapse and death (Pintar *et al*. 2003). There may also be a history of previous, milder episodes of weakness over the preceding few weeks. If weakness and a distended abdomen are seen in conjunction with pale mucous membranes at presentation, abdominal imaging (preferably ultrasound) should be performed immediately to look for free peritoneal fluid and intra‐abdominal masses, especially in the spleen. If effusion is present, then a sample can be obtained via abdominocentesis to determine if the fluid is haemorrhagic. Even if the peritoneal effusion is caused by a ruptured tumour, neoplastic cells are rarely seen on cytological evaluation of the fluid. Diagnostic tests should be performed to elucidate the cause of effusion, identify any haematological abnormalities which require intervention and identify metastasis prior to surgical intervention in cases of neoplasia. Blood abnormalities that may be present in cases with a haemoperitoneum consist of both regenerative and non‐regenerative anaemia, neutrophilia and thrombocytopenia (Scavelli *et al*. 1985 and Pintar *et al*. 2003). Thrombocytopenia occurs in 75–97% of cases of haemoperitoneum due to HSA (Hargis & Feldman 1991; Swardson *et al*. 1991; Pintar *et al*. 2003; Hammond & Pesillo‐Crosby 2008). Biochemical abnormalities are typically nonspecific (Pintar *et al*. 2003 and Culp *et al*. 2008). Coagulopathies are present in the majority of patients; disseminated intravascular coagulation (DIC) occurs in approximately 50% of dogs with HSA (Filppi *et al*. 1991; Maruyama *et al*. 2004 and Mischke *et al*. 2005)., Fine needle aspiration of any masses may be attempted, but can be non‐diagnostic, due to the vascular nature of many of these tumours and could cause further bleeding. Therefore, a preoperative diagnosis of the tumour may not be possible. Surgical resection is the best option for initial treatment of a bleeding mass, so it is reasonable to perform surgery to remove the mass and obtain a diagnosis through histopathological evaluation of the tumour tissue. If gross metastatic disease is present, however, surgery may not resolve the bleeding, so staging tests (3‐view thoracic radiographs, abdominal ultrasound and cardiac ultrasound) should be strongly advised prior to surgery. Thoracic radiographs are reported to have 78% sensitivity and 74% negative predictive value for detecting pulmonary metastasis of HSA when compared to post mortem findings (Holt *et al*. 1992). A non‐ruptured splenic mass is more likely to be benign (Cleveland & Casale 2016) but staging prior to surgery should still be advised.

Initial focus for the emergency clinician dealing with a haemoperitoneum is stabilization prior to surgery by re‐establishing the patient's effective circulating volume, maintaining their oxygen‐carrying capacity and arresting ongoing haemorrhage (Herold *et al*. 2008).

These patients are usually hypovolaemic secondary to the haemorrhage. Conventional fluid resuscitation (CR) can be used to replace these deficits and involves administration of an aliquot of fluid, which is approximately one‐quarter to one‐third of the estimated total blood volume (90 mL kg^−1^ in dogs and 60 mL kg^−1^ in cats), over 15–30 min. The patient's vital signs are reassessed following each aliquot and if still displaying signs of shock, the aliquot repeated. Evidence of stabilization on physical exam includes a normalization in blood pressure (90–160 mmHg), heart rate (80–140 BPM), respiratory rate (20‐40 BPM) and mentation (Rozanski & Rondeau 2002; Driessen & Brainard 2006; Hammond & Holm 2009). CR can lead to rapid redistribution of fluid to the interstitial space leading to oedema formation, hypothermia and may exacerbate bleeding by dislodging clots and diluting the circulating clotting factors. Another option specifically evaluated in canine cases of spontaneous haemoperitoneum is limited fluid volume resuscitation (LFVR) (Hammond & Holm 2009). This involves using the smallest possible volume of fluid to restore intravascular volume and minimize fluid extravasation into the interstitium. Typically, a colloid or hypertonic fluid is combined with isotonic crystalloids to achieve this goal using lower doses of fluids (5–10 mL kg^−1^) and re‐evaluating the patient's response. The blood pressure resuscitation endpoint with LFVR is lower than that of CR with values of 70 mmHg mean arterial pressure or 90 mmHg systolic arterial blood pressure being acceptable until definitive haemostasis is achieved. These values were derived as at a MAP of 60–70 mmHg, cerebral and renal blood flow are preserved (Hammond & Holm 2009). Invasive arterial blood pressure should be used to guide therapy with LVFR as non‐invasive measurements may overestimate blood pressures (Binns *et al*. 1995; Caulkett *et al*. 1998; Bosiack *et al*. 2010). Until definitive control of haemorrhage is achieved in these patients, small boluses of hypertonic saline and colloids are used to help resolve shock and perfuse the vital organs while the patient is being prepared for surgery. A prospective study comparing CR vs. LFVR for dogs with spontaneous haemoperitoneum showed that those receiving LFVR achieved stabilization faster than those receiving CR (Hammond *et al*. 2014). Disadvantages of LFVR include increased cost with colloids use, hypernatraemia due to hypertonic fluid and the need for rapid surgical control of haemorrhage once LVFR is employed to avoid organ dysfunction as the patient is being maintained at a lower blood pressure than compared to CR (Hammond *et al*. 2014).

A patient that is unresponsive to crystalloid or colloid fluid resuscitation, has evidence of severe haemorrhage or has elevated clotting times should be given a blood product (Herold *et al*. 2008; Culp & Silverstein 2015). Transfusion therapy is a treatment option that a clinician may employ in the pre‐operative, perioperative or post‐operative period as an effort to stabilize a case with a haemoperitoneum. Clinicians utilizing this therapy should aim to maintain the patient's haematocrit above 25% to stabilize signs of shock that are not responsive to fluid therapy and in the case of plasma transfusions to maintain clotting times within the normal range (Culp & Silverstein 2015). If blood products or a donor are not readily available, autologous blood transfusion (ABT) is another, albeit controversial, treatment option. The advantages disadvantages of ABT (Table 3) should be discussed with the owner. For routine transfusions, the blood product does not need to be administered warmed however if the clinician wants the product to be administered warmed, the blood can be warmed in a temperature‐controlled bowl (<39°C). Blood products should never be microwaved. Blood bags are connected to infusion sets which contain an in‐line micro‐filter (typically 170 micromilliliter pores). Sterility should be maintained at all times when connecting the bag to the fluid line. Ideally, the blood should be administered via gravitational flow however this may not be possible for smaller animals and infusion pump can be used in that case. The rate of transfusion will depend on the animal's cardiovascular status with the initial rate ideally being slow to observe any transfusion reactions. Transfusion of a single bag should be administered over 4 h to reduce the risk of bacterial contamination. The volume of blood to be transfused should be guided by goal directed therapy and the severity of anaemia, availability of blood products and the size of the patients. Several formulae have been described for dosing these products in dogs and cats. The two most accurate formulae in dogs include the following: (1) volume to be transfused (VT) = [(desired PCV – patient PCV/)donor blood PCV] x blood volume x body weight (kg) and (2) VT = required PCV% increased x 1.5 x body weight (kg) (Short *et al*. 2012). In studies in cats, the following formula used to predict the increase in PCV post transfusion was found to be most accurate: PCV% increase = VT (mL)/2 × body weight (kg) (Reed *et al*. 2014).

**Table 3:** Autologous blood transfusion

Advantages	Disadvantages
Decreased risk of transfusion reaction	Haemolysis
Decreased risk of inadvertent circulatory overload	Coagulopathy
Prevention of transfusion induced hypothermia	Microembolism of fat or air
Immediately available compatible blood	Potential spread of bacteraemia or sepsis
	Potential spread of malignancy

Conflicting reports exist in human literature as to the concern that ABT results in the dissemination of malignant cells (Burrows & Tartter 1982; Klimberg *et al*. 1986; Miller *et al*. 1991). Limited information is available in veterinary literature. Higgs *et al*. (2015) evaluated the outcome following ABT in 25 dogs secondary to thoracic or abdominal haemorrhage. In this study 32% of dogs needed an ABT as a result of a ruptured tumour, of those 75% had ruptured splenic masses. Seventeen of 25 dogs survived to discharge with 2/17 dying within 2 weeks of the ABT. Both dogs had a recurrence of haemoperitoneum (Higgs *et al*. 2015). Therefore if ABT is warranted it is important that the clinician discuss the benefits and risks associated so the owner may make an informed decision as to how to proceed.

The two methods for ABT include direct re‐infusion and cell salvage. As the latter requires specialized equipment and training it will not be discussed in further detail. Direct re‐infusion is the sterile collection of blood from the patient and re‐infusion into the same animal without processing and without the use of cell salvage device. While haemorrhage into a cavity is usually defibrinated within 45‐60 minutes, the addition of an anticoagulant is recommended with either method (Purvis 1995; Adamantos & Smith 2016).

Direct re‐infusion can be achieved either via a 2‐syringe technique (Robinson *et al*. 2016) or with a stopcock with or without the aid of underwater drainage set (Purvis 1995). In the 2‐syringe technique, blood is aspirated from the cavity into syringes containing acid citrate dextrose anticoagulant, transferred into a leur lock syringe attached to an infusion line and a 40‐micron blood filter and infused into the cephalic vein (Robinson *et al*. 2016). In the latter blood is aspirated from the cavity using an anticoagulant containing 60 mL syringe, stopcock and infusion line.

It is important the emergency clinician recognize patients with splenic masses and/or haemorrhagic peritoneal effusion may develop additional complications such as significant arrhythmias. Ventricular arrhythmias can be seen in both the perioperative and postoperative period and may be as a result of poor myocardial perfusion secondary to hypoxia, hypovolaemia, anaemia or a neurohormonal response associated splenic manipulation (Keyes & Rush 1994). Although arrhythmias can occur following splenectomies regardless of underlying splenic disease, it is much more likely associated with dogs who had ruptured splenic masses secondary to neoplasia (Marino *et al*. 1994). In one study dogs experiencing arrhythmias had a histopathological diagnosis of splenic HSA, haematoma and haemangioma in descending order (Keyes & Rush 1994). Ventricular arrhythmias resulting in haemodynamic instability may require treatment with an anti‐arrhythmogenic medication.

Part of the role of the emergency clinician in these cases also involves helping the owners make decisions regarding pursuing surgery for their pet. HSA is the most common splenic neoplasm in dogs and has a poor prognosis even with aggressive post‐operative therapy (Hammond & Pesillo‐Crosby 2008). Efforts have been made to help identify factors which may indicate a higher likelihood of HSA to help guide owners’ decisions in these difficult cases. Indicators of HSA in a 2016 study of dogs included preoperative anaemia, the presence of haemorrhagic ascites, thrombocytopenia and the need for a blood transfusion (Sherwood *et al*. 2016). The size or presence of focal as compared to those with multiple splenic masses may also have a bearing on prognosis with larger or more focal masses considered less likely to be malignant (Spangler & Kass 1997).

Prognosis following successful splenectomy depends on the underlying splenic disease. Long‐term survival for HSA remains poor; however, splenectomy can be curative with other tumours such as haemangioma and haematomas (Wendelburg *et al*. 2015). The median survival time for dogs with splenic HSA treated with splenectomy alone has been reported to be 86 days. In dogs that received chemotherapy (generally with doxorubicin‐containing protocols) post‐splenectomy, median survival times ranging from 125 days to 162 days have been reported. (Filppi *et al*. 1991; Ogilvie *et al*. 1996; Wood *et al*. 1998; Lana *et al*. 2007; Wendelburg *et al*. 2015; Matsuyama *et al*. 2017). In dogs, when haemoperitoneum is caused by some malignant tumours other than HSA (for example LSA and non‐angiomatous/non‐lymphomatous splenic sarcomas. In cases where there is not significant metastasis the prognosis is likely better and splenectomy alone can result in good survival, especially in cases of splenic marginal zone lymphomas (Gower *et al*. 2015; van Stee *et al*. 2015; Stefanello *et al*. 2011; Dervisis *et al*. 2017). Similarly, in cats, survival for those with neoplasia presenting with a haemoperitoneum was poor (Culp *et al*. 2010).

## Pathological fractures with bone neoplasia

Appendicular osteosarcoma is the most common primary bone tumour in dogs (Brodey & Riser 1969). Less common primary bone tumours include chondrosarcoma, fibrosarcoma, HSA, histiocytic sarcoma and synovial cell sarcoma (Dernell *et al*. 2007). The metaphyseal region of long bones is the most common site for osteosarcoma in dogs with the distal radius and proximal humerus being the most common sites (Knecht & Priester 1978). Metastasis to bones, either from osteosarcoma, or from primary tumours in other organs, can also occur. The most frequent sites for bone metastases include the vertebrae, ribs, humerus and the femur with haematogenous spread more common than extension to the lymph nodes for animals with osteosarcoma (Hillers *et al*. 2005). When evaluating canine patients, several types of neoplasia can metastasize to bone including HSA, mammary gland carcinoma, urogenital carcinomas, squamous cell carcinomas and histiocytic sarcomas. These metastatic bone lesions are seen less frequently than primary bone tumours (Liu *et al*. 1977; Trost *et al*. 2014). When metastasis is seen in long bones, it mainly affects the proximal metaphysis, and vertebral metastases are usually seen in the bodies of the thoracic and lumbar vertebrae, although the spinous processes of the thoracic vertebrae are occasionally affected. (Trost *et al*. 2014). In contrast, in cats the most common secondary bone tumours identified included fibrosarcoma, squamous cell carcinoma, LSA, HSA, rhabdomyosarcoma and meningioma (Lui *et al*. 1974).

Common clinical signs related to bone neoplasms include lameness and localized limb swelling (Dernell *et al*. 2007). Lameness occurs secondary to periosteal inflammation, microfractures or less commonly pathological fractures (PF) secondary to the expanding tumour effects on the bones’ density (Dernell *et al*. 2007). The femur is most commonly associated with PF and the radius least likely (Rubin *et al*. 2015). Diagnosis of a bone tumour is usually obtained via a radiograph of the affected limb where an osteolytic or osteoblastic pattern is usually noted (Thrall 1998; Dernell *et al*. 2007). An osteolytic pattern is characterized by destruction of the bone with little or no defensive response. Contrastingly, an osteoblastic pattern is defined with new bone formation and an increased radiographic opacity of the affected bone (Kealy *et al*. 2010). Lytic tumours are more likely to experience PF compared with osteoblastic lesions (Rubin *et al*. 2015). Osteomyelitis, including fungal and bacterial infections, is the most likely differential diagnosis. A bone biopsy can be performed for histopathological diagnosis and cultures if warranted. Bone biopsy carries the risk of causing PF but in the authors’ experience this risk is low. Thoracic radiographs are also recommended although fewer than 15% of dogs affected by osteosarcoma will have clinically detectable metastasis at the time of initial diagnosis (Dernell *et al*. 2007).

The greatest density of afferent nociceptors are found along the periosteal surface and within the medullary cavity of the bone. Thus, PF in both human and veterinary patients are associated with intense, persistent pain (Ehrhart *et al*. 2013). Additional discomfort can be attributed to chemical mediators from non‐neoplastic stromal cells which stimulate nociceptors and osteoclastic bone resorption (Goblirsch *et al*. 2006, Clohisy & Mantyh 2003; [LM1]). For the emergency clinician, pain management and temporary stabilization of the fractured limb are the most important issues in these patients while amputation surgery is arranged. Patients with gross metastasis at the time of diagnosis and treated with amputation alone have a mean survival time of 133 days (Spodnick *et al*. 1992; Boerman *et al*. 2012; Ehrhart *et al*. 2013).

A multimodal approach to pain control provides synergistic analgesia while concurrently lowering the total dose of each analgesic resulting in fewer side effects (Jin & Chung 2001). In general, pain control in the hospitalized patient can be provided at regularly timed intervals or as a constant rate infusion (CRI). CRI allows one to titrate the dose allowing better control of acute pain in a hospital setting. Intermittent administration of a non‐steroidal anti‐inflammatory drug with an opioid could be considered. Additionally, an N‐methyl‐D‐aspartate antagonist such as amantadine or ketamine can be used to help alleviate central sensitization. Under sedation or anaesthesia, ultrasound guided nerve blocks of the sciatic, femoral, radial and brachial nerves can provide additional pain control, however, specialized equipment and experience may limit the availability of these options (Bhoi *et al*. 2012).

Once a PF has occurred, it generally will not heal well since the edges of the bone surrounding the fracture are diseased. Therefore, amputation of the affected limb is generally considered to be the treatment of choice, but it is best to look carefully for evidence of gross metastatic disease before deciding on whether to perform an amputation. If visible metastasis is present, especially is there is diffuse pulmonary metastatic disease or areas of bone metastasis, then the prognosis following amputation is very poor, and amputation may not improve quality of life for long, or at all, in which case, the owners may elect to forgo amputation. Stabilization of the fractured limb can be accomplished under heavy sedation or anaesthesia and allows the application of thick layers of padding and a splint in order to prevent worsening of soft tissue trauma, improve patient comfort and assist in weight bearing if ambulation is attempted. Other treatment options for PF, such as limb sparing surgery, chemotherapy and radiation are generally considered to be ineffective and are outside the scope of this review.

## Conclusion

With an increase in the treatment options for cancer in small animal patients, veterinarians both in general practice and in the emergency setting can expect to see an increasing number of patients presenting with complications both from cancer itself and its treatment. Prompt recognition and appropriate therapy is required when managing these patients and management should include both a discussion with the owner about their goals and communication with a veterinary oncologist to maximize success. While few neoplasms can be fully cured in veterinary medicine, treatment of some of these oncological complications can be rewarding and prolong a good quality of life for the affected animals.

## Source of funding

There was no external funding for this report.

## Conflicts of interest

The authors report no conflicts of interest.

## Ethics statement

The authors declare human ethics approval was not needed for this study

## Contributions

KLT and LM conceived the review and with DH wrote the manuscript. All of the authors reviewed, revised and accepted the manuscript
